# Linezolid resistance in patients with drug-resistant TB and treatment failure in South Africa

**DOI:** 10.1093/jac/dkz206

**Published:** 2019-05-12

**Authors:** Sean Wasserman, Gail Louw, Limpho Ramangoaela, Garrick Barber, Cindy Hayes, Shaheed Vally Omar, Gary Maartens, Clifton Barry, Taeksun Song, Graeme Meintjes

**Affiliations:** 1 Wellcome Centre for Infectious Diseases Research in Africa, Institute of Infectious Disease and Molecular Medicine, University of Cape Town, Cape Town, South Africa; 2 Division of Infectious Diseases and HIV Medicine, Department of Medicine, University of Cape Town, Cape Town, South Africa; 3 Institute of Infectious Disease and Molecular Medicine, Department of Pathology, University of Cape Town, Cape Town, South Africa; 4 Jose Pearson Hospital, Eastern Province Department of Health, Port Elizabeth, South Africa; 5 National Health Laboratory Service, TB Laboratory, Port Elizabeth, South Africa; 6 Centre for Tuberculosis, WHO Supranational TB Reference Network, National Institute for Communicable Diseases, National Health Laboratory Service, Johannesburg, South Africa; 7 Division of Clinical Pharmacology, Department of Medicine, University of Cape Town, Cape Town, South Africa

## Abstract

**Objectives:**

Limited data exist on clinical associations and genotypic correlates of linezolid resistance in *Mycobacterium tuberculosis*. We aimed to describe mutations and clinical factors associated with phenotypic linezolid resistance from patients with drug-resistant TB at two public sector facilities in South Africa.

**Methods:**

Adults and adolescents with treatment failure (culture positivity ≥4 months) on a linezolid-containing regimen were retrospectively identified. Phenotypic resistance, as defined by a linezolid MIC >1 mg/L, was assessed for retrieved isolates using broth microdilution. Targeted sequencing of *rrl* and *rplC* was performed, irrespective of growth on subculture.

**Results:**

Thirty-nine patients with linezolid-based treatment failure were identified, 13 (33%) of whom had phenotypic or genotypic linezolid resistance after a median duration of 22 months (range = 7–32) of linezolid therapy. Paired MIC testing and genotyping was performed on 55 unique isolates. All isolates with phenotypic resistance (*n* = 16) were associated with known resistance mutations, most frequently due to the T460C substitution in *rplC* (*n* = 10); *rrl* mutations included G2814T, G2270C/T and A2810C. No mutations were detected in isolates with MICs at or below the critical concentration.

**Conclusions:**

Linezolid resistance occurred in a third of patients with drug-resistant TB and treatment failure. Resistance occurred late and was predicted by a limited number of mutations in *rrl* and *rplC*. Screening for genotypic resistance should be considered for patients with a positive culture after 4 months of linezolid therapy in order to optimize treatment and avoid the toxicity of ineffective linezolid therapy.

## Introduction

Drug-resistant TB has a major impact on health outcomes and costs in high-burden countries^1^ and is expected to increase over the next two decades.^2^ Linezolid, the prototype oxazolidinone, improved outcomes of drug-resistant TB in clinical trials.^3^^,^^4^ An individual patient data meta-analysis showed that linezolid use increased odds of treatment success 3-fold with a significantly lower mortality.^5^ Based on these data, WHO recommended linezolid as a preferred agent for all patients with drug-resistant TB in 2018.^6^ Linezolid therefore has an important role as an anti-TB agent and its introduction into national TB programmes will be scaled up.

Linezolid resistance has been reported in clinical isolates from a limited number of patients with drug-resistant TB and treatment failure.^4^^,^^7^ Limited evidence suggests that population-level resistance to linezolid may be increasing in TB programmes.^8^ Linezolid shares key binding sites and displays cross-resistance with other oxazolidinones, including promising new agents in clinical development, such as sutezolid^9^ and delpazolid.^10^ Mutations in genes encoding the 23S rRNA (*rrl*) linezolid peptidyl transferase centre (PTC) binding site and the L3 protein (*rplC*), which extends into the binding site, have been identified as the dominant molecular mechanisms underlying linezolid resistance from *in vitro* and clinical studies.^4^^,^^9–19^ There are limited data on the association between genotypic and phenotypic linezolid resistance in clinical isolates. The few published studies describing linezolid resistance in treatment programmes have not integrated MIC values with genotyping and have not explored important clinical parameters such as duration of linezolid exposure.^19^

There are two potential risk factors for linezolid resistance. First, linezolid dosing is frequently reduced due to mitochondrial toxicity,^20^ which may lead to suboptimal exposures for efficacy and resistance suppression, driving the selection of resistant mutants.^20^ Second, there are limited treatment options for drug-resistant TB and linezolid may be added to a failing or inadequate regimen, exacerbating the risk of acquired resistance.

A better understanding of the clinical predictors and genotypic correlates of linezolid resistance is critical to inform strategies to preserve this important anti-TB agent. We conducted a retrospective cohort study of patients with drug-resistant TB and linezolid-based treatment failure at two TB referral hospitals in South Africa with the following objectives: (i) to determine the prevalence of linezolid resistance in this at-risk population; (ii) to identify the mutations associated with phenotypic linezolid resistance in clinical *Mycobacterium tuberculosis* isolates; and (iii) to describe clinical factors associated with linezolid resistance.

## Patients and methods

### Setting and study population

Adult and adolescent patients (≥13 years old) with treatment failure on a linezolid-containing regimen were retrospectively identified from two public sector TB facilities in South Africa: Jose Pearson Hospital in Port Elizabeth and Brooklyn Chest Hospital in Cape Town. These facilities manage both inpatients and outpatients with drug-resistant TB and use linezolid routinely in their treatment regimens for pre-XDR-TB (defined as resistance to rifampicin and isoniazid, plus fluoroquinolones or second-line injectables) and XDR-TB (as for pre-XDR-TB, but with resistance to both fluoroquinolones and second-line injectables). Treatment failure, and eligibility for inclusion in the analysis, was defined as a persistently positive sputum culture for *M. tuberculosis* or culture reversion after a negative culture in a patient who had received at least 4 months of linezolid-based therapy for TB.

### Data sources

#### Clinical cases

Registers of patients with possible linezolid-based treatment failure are maintained by facility staff members; these medical records were screened by a study investigator. Clinical data were extracted and captured directly onto electronic case report forms in REDCap.^21^ The index TB episode was defined as receipt of continuous treatment (with <3 months’ interruption) for rifampicin-resistant TB. We quantified the number of likely effective agents in the regimen by applying a scoring system according to resistance profile, prior exposure and known clinical effectiveness [detailed in Table S1 (available as Supplementary data at *JAC* Online)].^22^

#### Microbiological data

Sputum culture results from routine testing performed at the study sites are linked to the National Health Laboratory Services (NHLS) database, which was used to identify *M. tuberculosis* isolates from identified cases. All previous isolates were requested from both local NHLS laboratories and from the National Institute of Communicable Diseases (NICD), which performs extended drug susceptibility testing (DST) on clinician request. Available isolates were shipped in original liquid culture bottles to the biosafety level-3 (BSL3) laboratory at the Institute of Infectious Disease and Molecular Medicine at the University of Cape Town for linezolid resistance testing.

### Linezolid resistance testing

#### Isolate selection and culture conditions

Subculture was done for all samples from the first batch of retrieved isolates (*n* = 57); in subsequent batches, only paired isolates (the earliest and most recent) from each patient with linezolid-based treatment failure underwent subculture (*n* = 46). *M. tuberculosis* isolates were initially cultured in the BACTEC Mycobacteria Growth Indicator Tube (MGIT) 960 system (Becton, Dickinson and Company, Sparks, MD, USA) according to the manufacturer’s instructions. Subsequently, 100 μL of each MGIT culture was inoculated onto Löwenstein–Jensen (LJ) medium slants (Becton, Dickinson and Company) and incubated at 37°C for 4–6 weeks with continuous aeration. Colonies were scraped from LJ slants with visible bacterial growth and 10% glycerol stocks were made. Subcultures were initiated by inoculating 100 μL of the 10% glycerol stock in 10 mL Middlebrook 7H9 broth (Sigma–Aldrich) supplemented with 0.2% (v/v) glycerol, 0.1% Tween 80 and 10% (v/v) OADC and were incubated at 37°C until an OD_600_ value of 1 was reached.

#### Determination of linezolid MIC

Phenotypic linezolid resistance was assessed by MIC determination using a resazurin microtitre assay.^23^ Two-fold serial dilutions of linezolid (range 64–0.0625 mg/L) were made in 7H9 medium supplemented with 0.1% casitone, 10% OADC and 0.5% glycerol in 96-well U-bottomed plates. The enriched 7H9 broth containing retrieved *M. tuberculosis* isolates was diluted 1:1000 and inoculated into the linezolid-containing plates. Plates were incubated at 37°C for 14 days before adding 20 μL of 0.025% (w/v) resazurin (Sigma–Aldrich), followed by incubation for an additional 24 to 48 h. The MIC value was defined as the lowest linezolid concentration that inhibited growth, indicated by a colour change from blue to pink. Positive (*M. tuberculosis* isolate only) and negative (7H9 medium only) controls were included for each assay. Phenotypic resistance was defined by an MIC >1 mg/L, the recognized critical concentration for linezolid.^24^

#### DNA extraction, PCR amplification and sequencing

DNA was extracted from all MGIT cultures, including those without growth on LJ slants, using the Chelex method.^25^ Primers were designed to amplify coding and flanking regions for *rrl*, as well as an 814 bp product covering *rplC* (Table S2). These targets were selected because they encode regions in or near the 23S rRNA binding site^26^^,^^27^ and have been associated with linezolid resistance in clinical and laboratory-generated *M. tuberculosis* isolates.^28^ We also planned to sequence *rplD* (which encodes a putative resistance target in the L4 protein)^10^ in isolates with MIC >1 mg/L and no detectable mutations in *rrl* and *rplC*, but this was not required. Primer design was based on the genome sequence of the *M. tuberculosis* H37Rv reference strain (http://genolist.pasteur.fr/TubercuList) and performed using Primer 3 software version 0.4.0 (http://bioinfo.ut.ee/primer3-0.4.0/).

PCRs were performed under the following thermocycling conditions: 15 min of denaturation at 95°C followed by 35 amplification cycles (each cycle = 94°C for 1 min, 62°C for 1 min and 1 min of extension at 72°C) and a final elongation step of 10 min at 72°C. Successful PCR amplification was confirmed by gel electrophoresis. PCR products underwent Sanger sequencing at Central Analytical Facilities, Stellenbosch University, South Africa. Mutations were detected using CLC Main Workbench, Version 7.7.3 (QIAGEN, CA, USA) by aligning the reference H37Rv strain (ATCC 27294) sequence to the sequence from the clinical isolates. Genotypic resistance was defined as the presence of single nucleotide substitutions in *rrl* or *rplC* previously identified to be associated with linezolid resistance,^28^ as well as newly identified polymorphisms in close proximity to the linezolid binding pocket and associated with elevated MICs.

### Analysis

MIC distributions of isolates that underwent phenotypic DST were plotted. We used bivariate analysis to compare demographic profile, treatment history and linezolid exposures between patients who developed linezolid resistance (phenotypic or genotypic) with those who did not. Wilcoxon rank-sum testing was performed for comparisons of continuous variables and *χ*^2^ tests for categorial variables. A Kaplan–Meier survival plot was constructed for time to the detection of linezolid resistance, censored for death, loss to follow-up (LTFU) and at 36 months post-linezolid initiation.

### Ethics

This study was approved by the University of Cape Town Human Research Ethics Committee (reference 805/2016).

## Results

### Linezolid MIC distribution and associated resistance mutations

**Figure 1.**
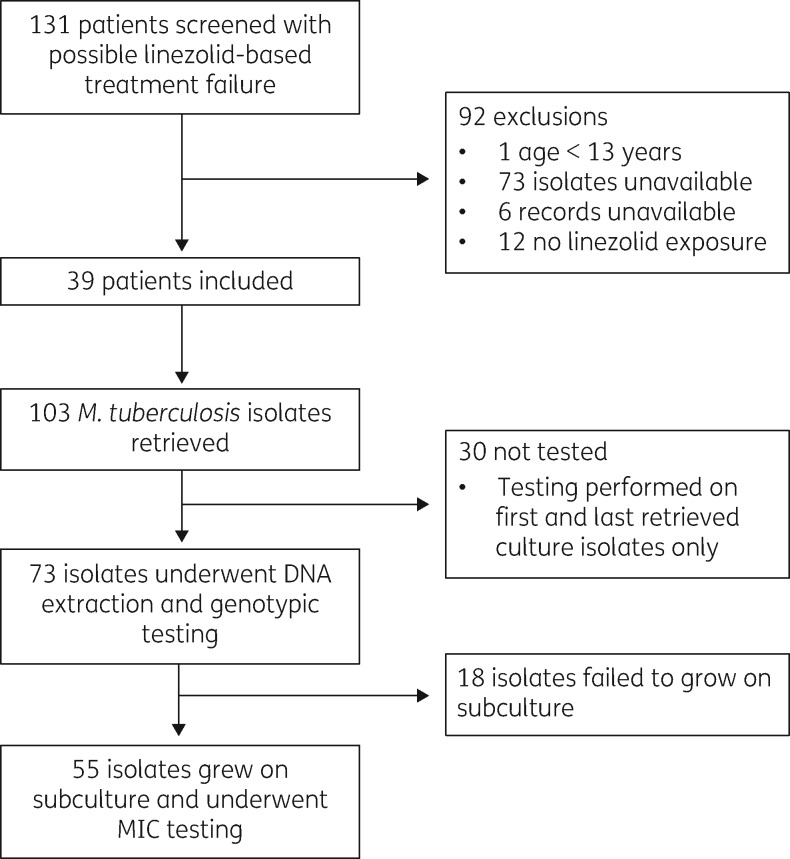
Flow diagram showing numbers of patients and isolates included.

**Figure 2.**
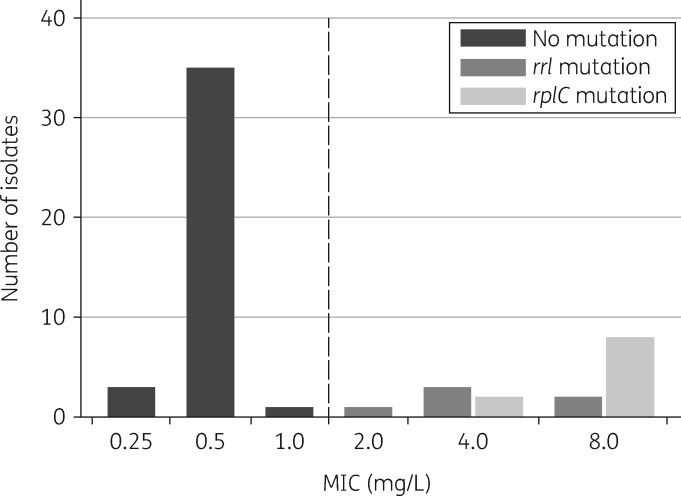
Distribution of *M. tuberculosis* linezolid MIC values for 55 clinical isolates with paired MIC and sequencing results. The vertical broken line represents the critical concentration value for linezolid (1 mg/L). None of the isolates had dual mutations.

We screened 131 patients with drug-resistant TB and suspected linezolid-based treatment failure (Figure 1); 103 *M. tuberculosis* culture isolates were available from 39 eligible patients (34 in Port Elizabeth and 5 in Cape Town) collected between May 2010 and September 2017. Paired MIC testing and genotyping were performed on 55 unique isolates that grew on subculture, demonstrating a clear bimodal distribution around the critical concentration of 1 mg/L (Figure 2). All isolates with MIC >1 mg/L (phenotypic resistance, *n = *16) were associated with known resistance mutations in either *rrl* or *rplC* (Table 1); conversely, no resistance-conferring mutations were detected in isolates with MICs at or below the critical concentration.

**Table 1.** Mutations in *rrl* and *rplC* with corresponding MIC values for all retrieved *M. tuberculosis* isolates that underwent phenotypic and/or genotypic resistance testing (*n = *73)

Participant ID	Isolate number	Duration on linezolid (months)^a^	Linezolid MIC (mg/L)	*rrl* mutation	*rplC* mutation
2007	XD00813360	10	8	**G2814T [2576]**	WT
1007	UH00774544	18	4	**(G2270T) [2032]**	WT
1008	UH00806598	25	8	WT	**T460C**
1010	YA00027930	12	2	**(G2270C) [2032]**	WT
1011	UH00751414	23	4	**G2814T [2576]**	WT
	UH00768146	24	4	**G2814T [2576]**	WT
1013	UH00760075	13	8	WT	**T460C**
	UH00830976	18	4	WT	**T460C**
1014	UH00812719	22	8	**G2814T [2576]**	WT
1015	UJ00479546	8	8	WT	**T460C**
	UJ00506756	10	8	WT	**T460C**
	UJ00519199	11	8	WT	**T460C**
1023	UH00754483	13	no growth^b^	A2384C [2146]	**T460C**
	TRL0118476	5	8	WT	**T460C**
1032	UH00873025	26	4	WT	**T460C**; G546A
1043	TRL0118350	10	8	WT	**T460C**
1050	UH00962529	23	8	WT	**T460C**
1057	UH00820877	25	no growth^b^	**A2810C [1942]**	WT

Nucleotide positions are given according to the sequence of *M. tuberculosis* strain H37Rv (GenBank accession number NC_000962.3) with corresponding *Escherichia coli* positions reported in square brackets. Mutations shown in round brackets were identified in the heteroresistant state. Bold formatting indicates resistance mutations.

a Linezolid exposure from time of initiation to collection of the isolate.

b No growth in LJ culture.

### SNPs associated with phenotypic resistance to linezolid

**Figure 3.**
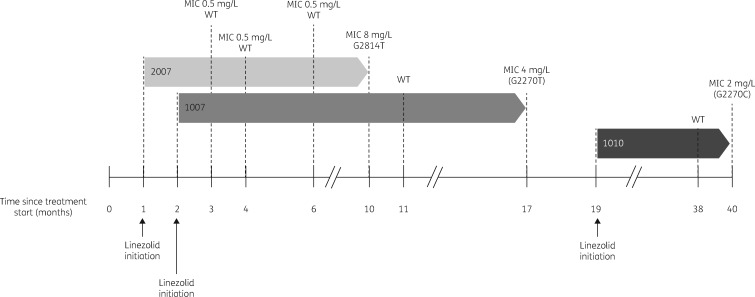
Timing of linezolid initiation and results of phenotypic and genotypic testing in relation to start of anti-TB therapy for three patients with sequential isolates demonstrating evolution of linezolid resistance. Inset numbers are study patient identifiers. Mutations shown in parentheses were identified in the heteroresistant state.

Sequencing of both *rplC* and *rrl* was done on 73 unique isolates (including isolates that failed to grow on subculture) from the 39 clinical cases with linezolid-based treatment failure. Mutations and corresponding MIC values from 13 patients (18 isolates) with phenotypic and/or genotypic resistance are listed in Table 1. Resistance mutations in *rplC* (*n* = 11) were detected more frequently than in *rrl* (*n* = 7); none of the isolates harboured dual resistance-conferring mutations. The G2814T substitution was the most frequently detected mutation in *rrl* (present in 4 out of 7 isolates), followed by point mutations in two isolates at position 2270 (G2270C/T) and one isolate with an A2810C mutation. The G2270C/T alleles were present with WT alleles as mixed populations from two unique patients and were not detected in strains recovered 1 month and 6 months earlier, respectively (Figure 3). We detected the following additional polymorphisms in *rrl*, which were not considered to represent resistance mutations due to distance from the PTC and because they were not associated with elevated MICs (MIC 0.25 mg/L for all): A2384G (*n = *3), A2384C (*n = *5), G2399A (*n = *1) and mixed G2399A/A2384C (*n = *3).

*rplC* resistance mutations (*n = *11) were exclusively due to T460C. We found a non-resistance-conferring polymorphism G546A (MIC 0.5 mg/L) in two isolates: as a single mutation in one isolate and mixed with T460C in another isolate with an elevated MIC. A GCC insertion at position 466 was identified in one isolate that failed to grow on subculture and consequently no MIC result was available; however, this insertion was not detected in a sequential isolate collected 6 months later.

### Clinical characteristics of the study population

Demographic and clinical characteristics of all included cases with linezolid-based treatment failure are shown in Table 2, disaggregated by the presence of linezolid resistance. Overall, 23/39 (59%) patients were HIV positive and the majority (28/39, 72%) had XDR-TB. There were 4 (median; IQR = 3.5–5) likely effective agents in addition to linezolid at the time of treatment failure; only 8/39 (21%) patients had isolates that were fully susceptible to fluoroquinolones, and bedaquiline and/or delamanid were included in the regimen for only 9/39 (23%) patients. Linezolid initiation was delayed for a median of 7 months (IQR = 2–17, range = 1–30) after the start of therapy for the index TB episode and was administered for a median of 16 months (IQR = 12–23, range = 5–44) until the last obtained culture result. The standard dose for linezolid was 600 mg daily, reduced to 300 mg daily in 20/39 (51%) patients.

**Table 2.** Clinical characteristics of patients with linezolid-based treatment failure

	Resistant, *n = *13	Susceptible, *n = *26	*P*
Age (years)	35 (30–45)	36 (28–42)	0.83
Male	6 (46)	13 (50)	0.82
Weight at treatment initiation (kg)	48 (39–62)^a^	45 (35–54)^b^	0.32
HIV-positive	7 (54)	16/25 (64)	0.54
Number of previous TB episodes	1 (1–2)	1 (1–2)	0.41
Baseline resistance pattern			
MDR	1 (8)	1 (4)	0.40
pre-XDR (injectable resistance)	1 (8)	5 (19)	
pre-XDR (fluoroquinolone resistance)	0 (0)	3 (12)	
XDR	11 (85)	17 (65)	
Delay in linezolid start after initiation of therapy (months)	8 (2–13)	3 (0–9)	0.24
Record of poor adherence	6/9 (67)	12/18 (67)	1.0
Linezolid dose reduction	6 (46)	14/23 (61)	0.39
Duration on linezolid (months)^c^	18 (10–23)	16 (12–21)	0.89
Number of other drugs	10 (9–11)	8 (7–10)	0.04
Number of likely effective drugs at time of treatment failure	4.0 (4.0–4.5)	4.0 (3.5–5.0)	0.95
Bedaquiline exposure^d^	3 (23)	9 (35)	0.46
Duration of bedaquiline exposure (months)^e^	5 (1–10)	10 (6–12)	0.19
Outcome within 48 months of study			
in care	6 (46)	8 (31)	0.55
died	6 (46)	9 (35)	
LTFU	0 (0)	2 (8)	
palliation	0 (0)	3 (12)	
unknown	1 (8)	3 (12)	

Data are presented as *n* (%) or median (IQR).

Resistant is defined as MIC >1 mg/L and/or presence of previously published resistance-conferring mutation.

a *n = *12.

b *n = *23.

c Defined as the time from linezolid initiation until the first culture showing linezolid resistance or the last culture obtained in those without linezolid resistance (*n = *39).

d The number of patients with bedaquiline exposure before the first culture showing linezolid resistance or the last culture obtained in those without linezolid resistance.

e Defined as the time from bedaquiline initiation until the first culture showing linezolid resistance or the last culture obtained in those without linezolid resistance (*n = *12).

### Clinical associations with linezolid treatment failure and resistance

**Figure 4.**
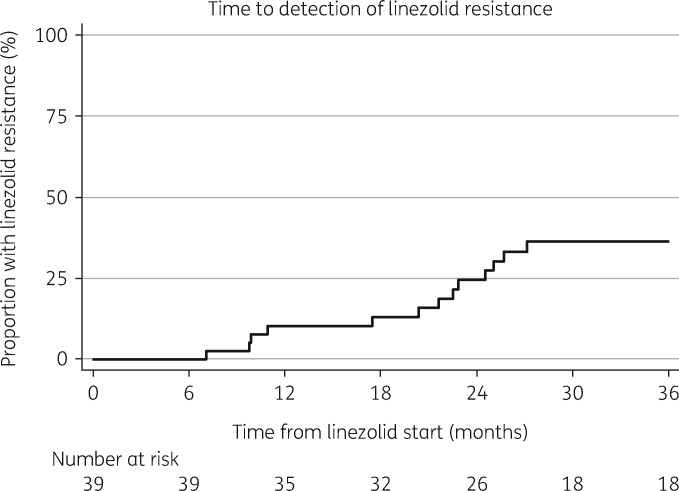
Kaplan–Meier plot showing time to detection of linezolid resistance, censored for death and LTFU (*n = *39).

Linezolid resistance was detected by either phenotypic or genotypic methods in 13/39 (33%) patients with linezolid-based treatment failure. The earliest detected occurrence of resistance was 7 months after initiating linezolid, with the latest at 27 months (Figure 4). Neither linezolid dose reduction (*P* = 0.39) nor overall duration (18 months for those with resistance versus 16 months without; *P* = 0.89) were associated with linezolid resistance (Table 2). Bedaquiline exposure prior to treatment failure did not appear to be protective for linezolid resistance in this cohort; 3/13 (23%) with resistance versus 9/26 (35%) without resistance received bedaquiline; *P = *0.46. The only significant difference between those with and without resistance on bivariate analysis was the total number of anti-TB drugs received, which was higher in the group with linezolid resistance (median 10 versus 8 drugs; *P = *0.04). There was a trend towards a longer delay in linezolid initiation in the group with resistance (8 months versus 3 months), but this was not significant (*P = *0.24). Overall mortality was 38% (*n = *15) during the 42 month observation time.

## Discussion

We retrospectively identified 39 patients with drug-resistant TB and linezolid-based treatment failure from two geographically distant treatment facilities in South Africa. Most patients had XDR-TB and were unable to obtain early access to new anti-TB agents. Consequently, background drug regimens at the time of linezolid introduction were likely suboptimal resulting in effective linezolid monotherapy for prolonged periods of time. Linezolid resistance was detected in over 30% of individuals and only after a median of 18 months of exposure, which is somewhat surprising given the highly conducive conditions for resistance selection. In one respect this observation is reassuring, because it confirms linezolid’s high barrier to resistance seen under similar conditions in a clinical trial,^4^ as well as *in vitro* where very low mutation frequencies are achieved relative to other anti-TB drugs.^9^^,^^14^ However, our finding also illustrates a fundamental reality underlying the large-scale expansion of linezolid for TB treatment: despite its high barrier to resistance, with sufficient selection pressure the emergence of linezolid resistance in TB treatment programmes is inevitable. Of concern, there have been suggestions of a trend towards increasing population-level resistance in countries with a long history of linezolid use;^8^ and linezolid resistance has been associated with the Beijing genotype,^18^ the dominant circulating *M. tuberculosis* strain in the Eastern Cape Province^29^ where most of our cases were identified. Increased vigilance and active surveillance are clearly needed as linezolid is introduced into national TB treatment programmes.

Published data on linezolid resistance from patients with TB are scarce; in a literature review we identified nine studies that investigated linezolid resistance in clinical isolates, reporting on a total of 24 unique patients. Our study, involving 39 patients with linezolid-based treatment failure, likely provides the largest and most detailed series linking MIC values with molecular testing and clinical and treatment data. There are several notable findings that build on existing knowledge in this area.

Our strategy to perform targeted sequencing of *rrl* and *rplC* was based on the mechanism of linezolid action and observations from clinical and *in vitro* reports. It is unsurprising that mutations in 23S rRNA, particularly in proximity to the PTC binding site, predictably led to MIC elevations and clinical resistance. The most frequently reported mutation in *rrl*, the G2814T nucleotide substitution,^4^^,^^9^^,^^19^^,^^30–32^ was detected in isolates from more than half of patients with linezolid resistance and *rrl* mutations, and associated with MIC values of up to 8 mg/L. We also detected G2270C/T mutations in isolates from two unique patients, which, to our knowledge, is the first report in clinical strains. These mutations were associated with lower MICs (2–4 mg/L) in our cohort (as well as in previous *in vitro* studies^14^^,^^17^), which may be related to the position outside the PTC and the fact that in both cases the mutations were identified in the presence of the susceptible allele (heteroresistant state). We did not detect the resistance allele in prior isolates from either of these patients (collected 1 month and 6 months earlier, respectively), suggesting the evolution of linezolid resistance with ongoing selection pressure.

The third *rrl* mutation we identified, A2810C, has been previously described in isolates from patients with treatment failure.^30^^,^^31^ Although we were unable to determine the MIC associated with this mutation, this nucleotide substitution is in relative proximity to the PTC and its previous detection in isolates with phenotypic resistance suggest that it could confer a linezolid resistance phenotype.

Overall, the T460C mutation in *rplC* was the underlying cause for linezolid resistance in the majority (7/13, 54%) of our cases; its dominance has also been noted in other settings.^9^^,^^12–14^^,^^17^^,^^19^ This mutation results in an amino acid exchange from cysteine to arginine at position 154 in the L3 protein that extends into the linezolid binding site,^27^ resulting in MIC ranges of 2–32 mg/L.^4^^,^^9–19^ The *rplC* T460C mutation has been associated with lower MICs and a lower fitness cost^9^ than mutations affecting 23S rRNA. Interestingly, in our cohort, the converse was found, with higher MICs linked to *rplC* mutations; this has also been described for *in vitro* mutants,^14^^,^^17^ reinforcing the importance of this key mechanism for linezolid resistance. It is difficult to interpret the significance of the GCC insertion at position 466 found in one isolate because we were unable to determine the MIC; this has not been previously associated with linezolid resistance, including in bacteria other than *M. tuberculosis*, and is likely not to be resistance-conferring.

Genotyping had excellent accuracy and discriminative value for predicting phenotypic resistance (MIC >1 mg/L) in the 55 isolates with both sequencing and MIC results. It appears that sequence mixes in *rrl* and *rplC* have low diversity and there are a limited number of mutations that underlie linezolid resistance. This raises the possibility of translation into rapid molecular diagnostics, which are needed to support linezolid rollout into national TB programmes where phenotypic testing is not widely available. Furthermore, molecular testing could be class-based because of cross-resistance with other oxazolidinones^9^^,^^10^^,^^13^ and has the advantage of detecting low-frequency mutations supporting early identification of resistance.

We found that the number of background agents to which patients were exposed during the index TB episode was significantly higher amongst those with linezolid resistance. This could reflect a tendency of clinicians to add more drugs to failing regimens, leading to a paradoxically higher risk of linezolid monotherapy with more background drugs. The observed trend in longer delays to linezolid initiation in patients with linezolid resistance supports this. Although there is no direct cross-resistance between linezolid and other anti-TB drug classes, one study has demonstrated an association between linezolid MIC elevations and the use of other second-line anti-TB drugs, specifically fluoroquinolones and kanamycin.^8^ This may be due to induction of efflux pump expression from antimycobacterial drug exposure, which initiates a pathway leading to subsequent high-level mutation-related resistance.^33^ Regardless of the presence of linezolid resistance there was an extremely high mortality amongst this cohort of patients with linezolid-based treatment failure. This emphasizes the need for early inclusion of new drugs such as bedaquiline to strengthen treatment regimens and reduce the risk of treatment failure^34^ and mortality.^35^

Our retrospective study had a number of important limitations. By definition, we had to rely on data that were collected in the clinical service and not originally intended to address the aims of this study. We therefore had to accept risks of major biases when describing and comparing patients with linezolid-based treatment failure. We are confident, however, that our strategy to screen and identify cases from hospital registers was sufficiently rigorous to avoid excluding important outliers. The accuracy of clinical data extracted from medical records was imperfect and also may have influenced the robustness of our findings, particularly in relation to anti-TB drug exposures and assessment of regimen effectiveness. However, the key parameters of linezolid duration and bedaquiline use were well documented. A substantial proportion of isolates were either not available from local laboratories (due to being lost or discarded; 73/131 screened patients, 56%) or were not viable on subculture (18/73 retrieved isolates, 25%). This has two potential consequences. First, sequential isolates demonstrating the transition to linezolid resistance were not available for the majority of included patients. The time-to-event analysis could therefore have overestimated the delay in the development of linezolid resistance. Second, isolates with linezolid resistance may be associated with fitness cost^9^ and failure to grow, biasing our results. To address this, we sequenced all isolates, regardless of culture viability, and found a resistance mutation in only two isolates without MIC data. Sanger sequencing itself has imperfect sensitivity, particularly for the detection of mixed strain genotypes;^36^ it is possible that we may have identified additional or novel mutations using next generation sequencing,^37^ an approach that should be considered in future studies.

In conclusion, we have shown that linezolid resistance occurred in a third of patients from this high-risk cohort in South Africa. Phenotypic resistance was detected late and was predicted by a limited number of mutations in *rrl* and *rplC*. Screening for genotypic resistance should be considered for patients with a positive culture after 4 months of linezolid therapy in order to optimize treatment and avoid the toxicity of ineffective linezolid therapy.

## Supplementary Material

dkz206_Supplementary_Data
