# Supplementary material for: Linezolid resistance in patients with drug-resistant TB and treatment failure in South Africa
Source: J Antimicrob Chemother. 2019 May 12;74(8):2377–84. doi: 10.1093/jac/dkz206 (PMC6640298; doi:10.1093/jac/dkz206)
Supplement: dkz206_Supplementary_Data [file dkz206_supplementary_data.docx]

**Supplementary data**

**Table S1.** Rules for rating likely effectiveness of drugs in background regimen

| **Drug** | **Context/rationale** | **Score** |
| --- | --- | --- |
| Pyrazinamide | Baseline resistance in MDR/XDR TB is ~60%^1^ | 0.5 |
| Fluoroquinolones | XDR or pre-XDR with FQ resistance  All other circumstances | 0  1 |
| Injectable agents | XDR or pre-XDR with injectable resistance  All other circumstances | 0  1 |
| Ethambutol | Baseline resistance in MDR/XDR TB is ~45%^1^ | 0.5 |
| Isoniazid | inhA mutation  Dual inhA/katG mutations or katG mutation alone | 0.5  0 |
| Ethionamide | Dual inhA/katG mutations or inhA mutation alone  All other circumstances | 0  1 |
| Clofazimine | If previous XDR or pre-XDR-TB treatment (likely exposed)  No previous XDR or pre-XDR-TB treatment (no exposure) | 0.5  1 |
| PAS | If previous XDR or pre-XDR-TB treatment (likely exposed)  No previous XDR or pre-XDR-TB treatment (no exposure) | 0.5  1 |
| Terizidone | If previous MDR-TB treatment (likely exposed)  No previous MDR-TB treatment (no exposure) | 0.5  1 |
| Macrolides | No clinical efficacy | 0 |
| Amoxicillin-clavulanate | No clinical efficacy | 0 |
| Bedaquiline | No previous exposure | 1 |
| Rifabutin | No previous exposure, and susceptible by genotyping | 1 |
| Delamanid | No previous exposure | 1 |

Molecular and/or phenotypic drug susceptibility testing results from routine testing were available for rifampicin, isoniazid (including the presence of *inhA* and *katG* mutations), fluoroquinolones, and second-line injectables for at least one isolate from each patient.

**References**

**1** Ismail NA, Mvusi L, Nanoo A *et al*. Prevalence of drug-resistant tuberculosis and imputed burden in South Africa: a national and sub-national cross-sectional survey. *Lancet Infect Dis* 2018; **18**: 779–87.

**Table S2.** Primer sets used for the amplification and sequencing of *rplC,* *rrl* and *rplD*

| Primer | Sequence 5’ to 3’ | Fragment length (bp) |
| --- | --- | --- |
| rplC F | CACAAGCGGTTGATCGACAT | 814 |
| rplC R | GCGTCTTGACGTCGATTTTG |  |
| rrl (1) F | AGTTGGCCACCAACACACTG | 944 |
| rrl (1) R | TGGGTCGCCCTATTCAGACT |  |
| rrl (2) F | GGTTAACCCGTGTGGGGTAG | 1101 |
| rrl (2) R | TTCTTGGCAGCAGAGGATCA |  |
| rrl (3) F | CGATGGACAACGGGTTGATA | 1016 |
| rrl (3) R | GGCGCCTCCGTTACATTTTA |  |
| rrl (4) F | CGAAATTCCTTGTCGGGTAAG | 1017 |
| rrl (4) R | ACGGATGTGGTTGCGAGTTT |  |
| rplD F | TTGGTGCATAAGGTCGATGC | 941 |
| rplD R | TGACGGCAAAAATCTTCTCG |  |
